# A Modern Roman‐Inspired Concrete with Daytime Radiative Cooling Capacity

**DOI:** 10.1002/advs.202511691

**Published:** 2025-09-12

**Authors:** Jorge S. Dolado, Guido Goracci, Ghizlane Moutaoukil, Ridwan O. Agbaoye, Miguel Beruete, Alicia E. Torres‐García, Laura Carlosena, Achutha Prabhu, Jose A. Ibáñez, Nick Adams, Nicole van Lipzig, Karen Allacker

**Affiliations:** ^1^ Centro de Física de Materiales (CFM) CSIC‐UPV/EHU) San Sebastián 20018 Spain; ^2^ Donostia International Physics Center (DIPC) San Sebastián 20018 Spain; ^3^ Department of Electrical Electronic and Communications Engineering Public University of Navarre (UPNA) Pamplona 31006 Spain; ^4^ Institute of Smart Cities (ISC) Public University of Navarre (UPNA) Pamplona 31006 Spain; ^5^ Department of Engineering Public University of Navarra (UPNA) Pamplona 31006 Spain; ^6^ TECNALIA, Basque Research and Technology Alliance (BRTA) Derio 48160 Spain; ^7^ KU Leuven, Faculty of Engineering Science Department of Architecture Kasteelpark Arenberg 1 – box 2431 Leuven 3001 Belgium; ^8^ KU Leuven, Department Earth and Environmental Sciences Celestijnenlaan 200E Leuven 3001 Belgium

**Keywords:** building energy, CO_2_ footprint, concrete, global warming, radiative cooling technology, urban heat island effect

## Abstract

Addressing global warming through the modernization of buildings and urban areas is a major challenge. Passive daytime radiative cooling (PDRC) materials offer potential solutions, but none have effectively replaced concrete's dominant role in urban environments. Here, a Roman‐inspired concrete with PDRC capabilities is presented, combining high solar reflectance (≈0.95) and long‐wave infrared (LWIR) emittance (≈0.91). It delivers cooling powers over 45 W m^−^^2^ under average solar intensities of 850 W m^−^^2^ without a convection shield. On hot days (above 30°C), it stays 2°C cooler than the surrounding air under solar irradiance up to 985 W m^−^^2^. Simulations predict this concrete can reduce energy use and CO_2_ emissions by ≈50% in hot regions and lower urban surface temperatures by up to 10°C during heat waves. This breakthrough offers a cheap, scalable and sustainable solution for energy efficiency and climate resilience.

## Graphical abstract

Coolcrete, inspired by ancient Roman concrete, is a new cementitious material with photonic properties enabling passive daytime radiative cooling. Made from quicklime and zeolites, it reduces cooling energy demand, lowers CO_2_ emissions, and may eliminate urban heat islands. Affordable and scalable, it integrates with current construction practices, offering a sustainable solution to urban environmental challenges.

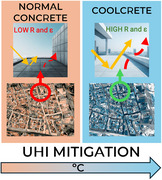

## 1 Introduction

In recent decades, the combined effects of human actions on our planet have resulted in global warming and the emergence of the urban heat island phenomenon.^[^ ^1^ ^]^ According to the Urban Climate Change Research Network, there is a projected increase in average urban temperatures by ≈4°C by 2050.^[^ ^2^ ^]^ This temperature rise brings about significant health risks for individuals who are exposed to prolonged sunlight, who may suffer from heat‐related illnesses like heatstroke, as well as mental health issues like anxiety and depression^[^ ^3^ ^]^.

In this regard, passive daytime radiative cooling (PDRC) materials^[^ ^4^, ^5^ ^]^ surely present the most suitable solution. These materials have the ability to reflect sunlight and release heat into the cold expanse of the universe through a specific atmospheric window (wavelengths approximately from 8 to 13 µm) without the need for any energy consumption.

Although PDRC ability has been demonstrated by diverse materials, like nanophotonic thin films,^[^ ^6^, ^7^ ^]^ polymeric nanotextiles,^[^ ^8^, ^9^, ^10^, ^11^ ^]^ nanocellulose,^[^ ^12^ ^]^ porous polymer coatings,^[^ ^13^, ^14^, ^15^ ^]^ glass,^[^ ^16^ ^]^ glass‐polymer hybrid metamaterials^[^ ^17^ ^]^ and inorganic hollow microparticle composites^[^ ^18^, ^19^, ^20^ ^]^ these solutions can seldom replace the dominant presence of cement‐based materials in urban areas. Concrete and cement‐based materials stand as the cornerstone of the burgeoning urban landscape. It not only serves as the foundational element of our evolving world but also reigns supreme as the preeminent synthetic construction material. Given their prevalence, it is not surprising that the idea of altering cement's optical properties to enable PDRC has recently garnered increasing attention from the scientific community,^[^ ^21^, ^22^, ^23^, ^24^, ^25^ ^]^ though without much success so far. To date, the best performance has been obtained by using cementitious armors^[^ ^26^ ^]^ and paints^[^ ^27^ ^]^ based on BaSO_4_ nanoparticles, which allow cementitious interfaces with building compatibility. In fact, no studied cementitious material apart from^[^ ^26^ ^]^ has met the practical threshold of reflecting over 94% of sunlight^[^ ^6^ ^]^ needed for meaningful daytime radiative cooling. In this work, we address this challenge by developing a novel cementitious composite with tailored composition and microstructure designed to enable PDRC performance, while preserving full compatibility with current construction and concrete technologies.

## 2 Results and Discussion

### 2.1 PDRC Concrete Engineering

**Figure 1:**
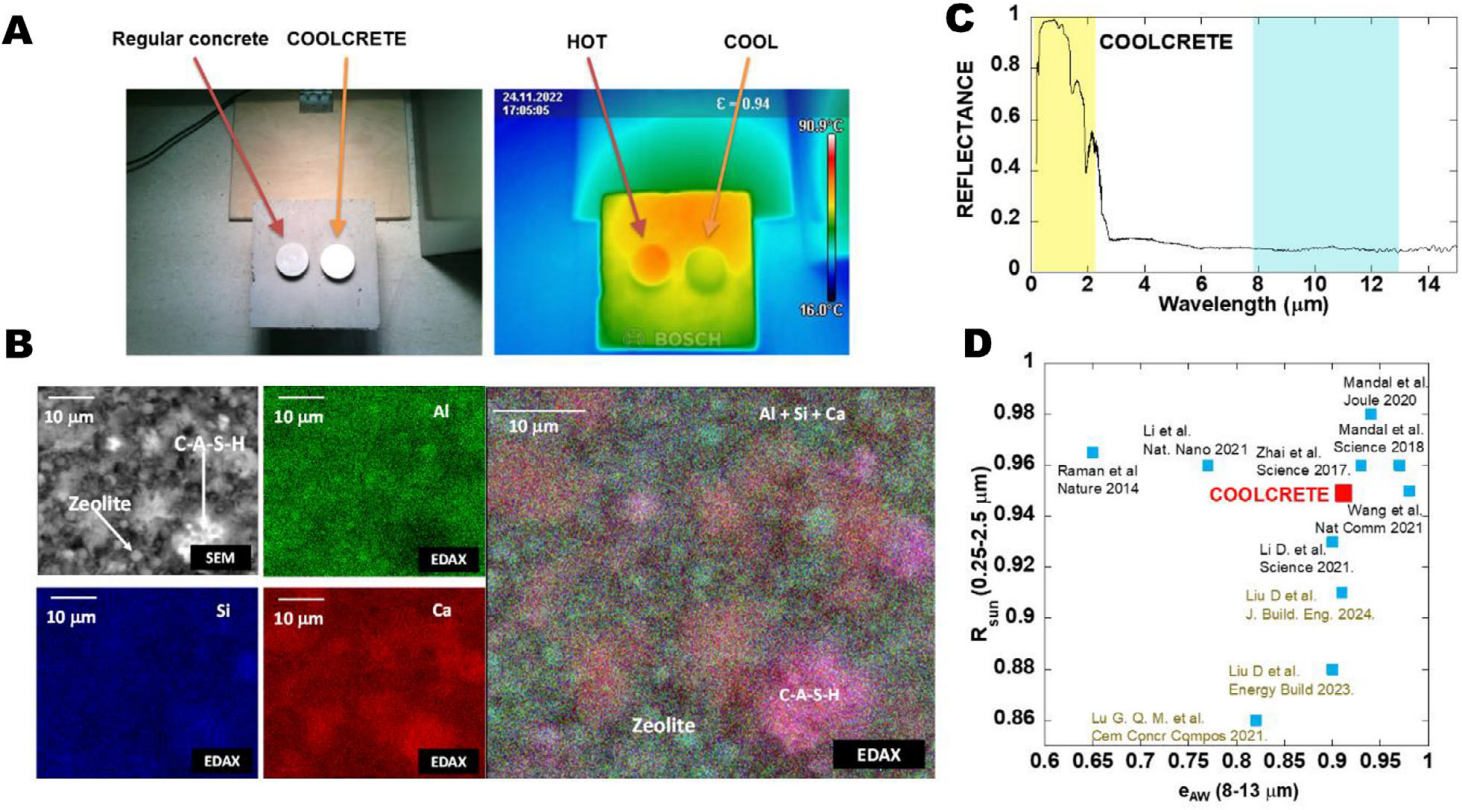
A modern Roman concrete with daytime radiative cooling ability. A) Optical and infrared images of regular concrete and PDRC concrete under an irradiance of 750 W m^−2^. B) Multi‐scale structure as observed by SEM and EDAX analysis: green regions indicate higher density of Al atoms (coming from the zeolites), red regions denote higher density of Ca atoms (coming from unreacted CaO and calcite), and purple regions indicate high presence of Ca and Si atoms (coming from calcium silicate hydrate (CSH)). C) Reflectance of our modern Roman mortar (coolcrete) with the solar range highlighted in light yellow and the atmospheric window in light blue. D) Comparison between the solar reflectances and LWIR emittances of coolcrete with other values of the state of the art. The values displayed in greenish‐brown are related to cement‐based materials.

In response to the aforementioned challenge, we engineered concrete (**Figure** **1**A) by a complete revision of its composition and structure that enables PDRC performance. Our educated optimization (from hereafter called coolcrete) has resulted in a sort of modern Roman‐inspired hydraulic mortar, based on the reaction of some specific pozzolanic materials with quicklime CaO in water (a process known as hot mixing).^[^ ^28^, ^29^ ^]^ There are two main salient particularities with respect to traditional ancient Roman Mortars: First, the used pozzolanas are zeolites (microporous crystalline structures) instead of the volcanic ashes (vesicular glassy structures) typically used in Roman Mortars. Second, small amounts of alite, Ca_3_SiO_5_ (C_3_S in cement chemistry notation), the most important phase of Ordinary Portland Cements (OPC), can be added to accelerate the hardening process and improve the mechanical properties (see Experimental Section). In fact, the production method of our coolcrete is simple, cheap, and fully compatible with current construction and concrete technology.

The key concept is the addition of enough zeolite content so that it can partially react with quicklime to produce a cohesive binder (a calcium‐aluminate‐silicate‐hydrated gel (C─A─S─H)), while a significant portion remains unreacted. In fact, as shown by SEM and EDAX analysis (Figure 1B), confocal Raman analysis (Figure S1A, Supporting Information) and XRD analysis (Figure S1B, Supporting Information), the overall microstructure of our engineered cementitious composite contains many undefective zeolites (≈51% by weight of the crystalline phases), which remain trapped in a porous tobermorite‐like structure that appear from the pozzolanic reactions between the aluminosilicate source (zeolite) and portlandite (the hydrated lime (CaO+H_2_O → Ca(OH)_2_))

(1) $$\begin{array}{ccc} \mathrm{xCa}{\left(\mathrm{OH}\right)}_{2} & + & \mathrm{yA}l_{2}O_{3}\mathrm{zSi}O_{2}+\left(n-\;x\right)H_{2}O\;\to \left(\mathrm{CaO}\right)x\cdot \\ & & \left(Al_{2}O_{3}\frac{}{}\right)y\cdot \left(\mathrm{Si}O_{2}\right)z\cdot \left(H_{2}O\right)n\;\\ & & \left(C-A-S-H\;\mathrm{gel}\;\right) \end{array}$$

Minor amounts of calcite (CaCO_3_) coming from the carbonation of the sample could also be found, the same as unreacted C_3_S when added in the formulation and as revealed by XRD experiments (Figure S1B, Supporting Information). Likewise, the porosity of our cementitious composite (Figure S1C, Supporting Information) is sufficiently refined to favour Mie scattering,^[^ ^30^ ^]^ something which has been recognized as a crucial factor for enhancing the radiative cooling capacity.^[^ ^31^ ^]^ All these phases (zeolites, C_3_S, tobermorite, and CaCO_3_), but mainly the zeolites, exhibit excellent properties for daytime radiative cooling properties (Figure S2A,B, Supporting Information), because they concurrently exhibit very high solar reflectance (R_sun_) and high emissivity in the atmospheric window (e_AW_). In other words, the deliberate incorporation of a large fraction of unreactive zeolites serves as a structural strategy to transform traditional Roman mortars^[^ ^28^, ^29^ ^]^ into modern versions with enhanced radiative cooling capabilities.

Altogether, the engineered cementitious composite efficiently backscatters sunlight and emits thermal radiation (Figure 1C). Remarkably, this modern Roman‐inspired cementitious composite shows exceptional values for R_sun_ (≈0.95) and e_AW_ (≈0.91). These values, especially the solar reflectance, clearly exceed the values found in other cement‐based composites, and are on par with state‐of‐the‐art PDRC designs^[^ ^4^, ^32^ ^]^ (see Figure 1D)

**Figure 2:**
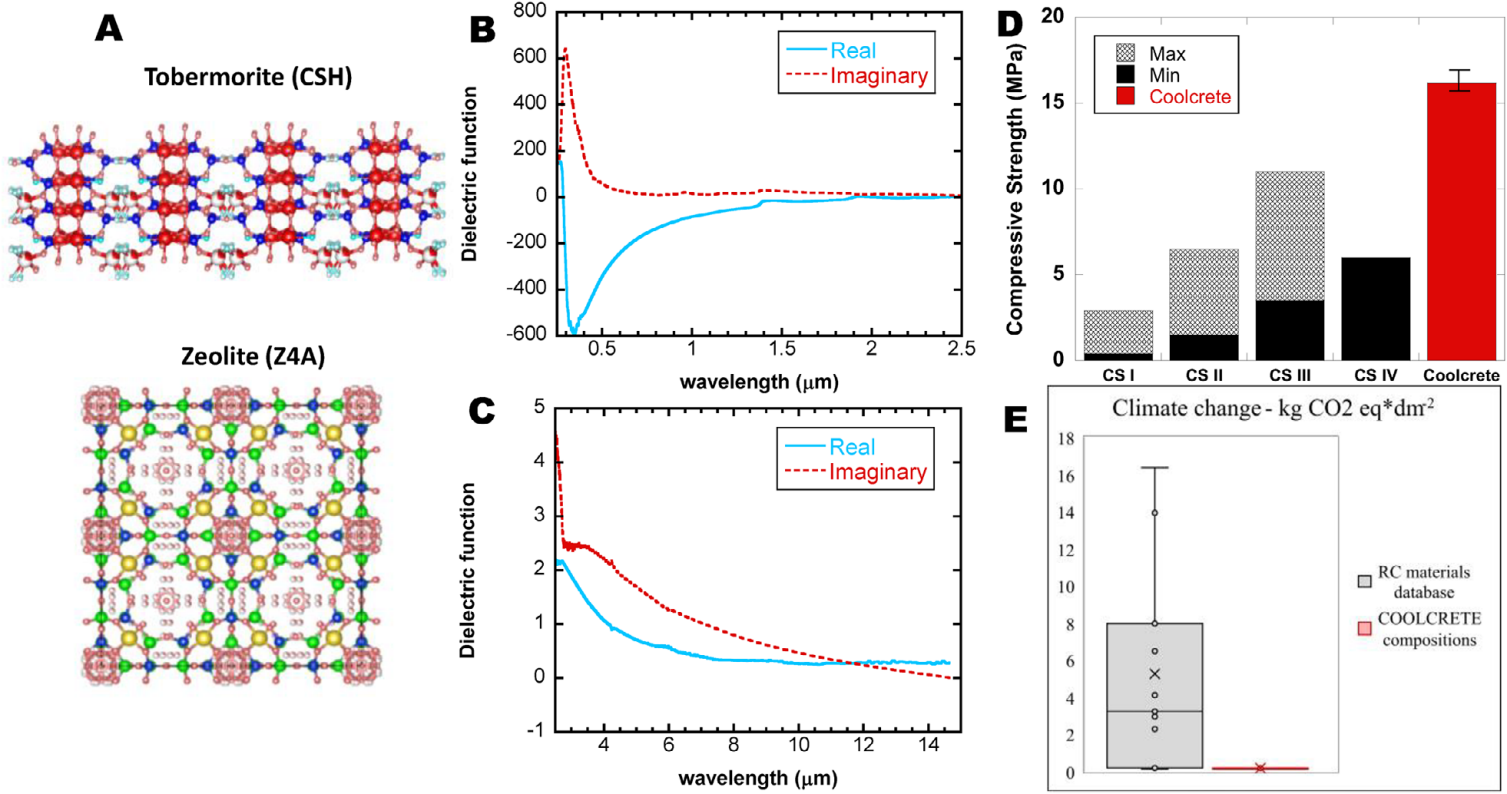
Photonic, mechanical, and environmental properties. A) Atomic structure of tobermorite (a mineral analog to CSH gel) and zeolite. B) Dielectric function of coolcrete in the solar domain (0.25–2.5 µm). C) Dielectric function of coolcrete in the NIR and IR domains. D) Compressive strength after 28 days of curing of coolcrete (16.6 ± 0.7 MPa) in comparison to standardized categories of masonry mortars, where 0.4 MPa < CSI < 2.5 MPa, 1.5 MPa < CSII < 5 MPa, 3.5 MPa < CSIII < 7.5 MPa, and 6 < CS IV; E) CO_2_ footprint of a tile made of our coolcrete (and rest of family members) in comparison to a regular concrete tile with radiative cooling coatings.

The optimal electromagnetic properties of the cementitious composite can be ascribed to those of the major phases, tobermorite^[^ ^33^ ^]^ and zeolites^[^ ^34^ ^]^ (**Figures** **2**A; S2A, Supporting Information). The complex dielectric function of the sample (Figure 2B,C) was obtained from the reflectance measurements by applying the Kramers‐Kronig relations following the scheme used in A. E. Torres‐García et al.,^[^ ^35^ ^]^ exhibiting very negative values of the real component of the dielectric function within the solar range (0.25–2.5 µm). This behavior clearly stems from multiple Mie scattering processes^[^ ^30^ ^]^ arising from air‐filled pores embedded within the higher‐index cement matrix, which act as low‐permittivity scatterers, inducing localized resonant scattering and a dispersive polarization response analogous to Lorentz‐type oscillators.^[^ ^22^, ^30^, ^31^, ^35^ ^]^ This phenomenon explains the high sun reflectance ability of the engineered cementitious composite. Interestingly, the sample behaves very absorptive in the infrared regime with low but positive real and imaginary components of the dielectric function, guaranteeing an appropriate emissivity within the AW.^[^ ^35^ ^]^

### 2.2 Properties of Coolcrete

The radiative cooling concrete is not intended to be used as structural concrete, because its effect is essentially superficial, and cementitious layers below a few centimetres might suffice to guarantee the performance. However, a key property of cement‐based materials is their ability to withstand loads. In that sense, the compressive strength of the coolcrete after 28 days of curing (Figure 2D) clearly exceeds the values of the different categories (CS I, CS II, CS III, and CS IV) specified in the European Standard EN‐998‐1 for mortars for masonry. Still better mechanical properties (comparable with standard strength concrete grade C20) can be achieved by adding small amounts of alite at the cost of only a slight reduction of the photonic response (Figure S2B,C, Supporting Information). Besides, the good ratio between the mechanical properties of our coolcrete to weight allows their use in multiple components and shapes (plain roofs, tiles, facades, etc).

Another important feature of our engineered coolcrete is its hydrophobic surface, which effectively repels waterborne dirt (see Figure S2C, Supporting Information), contributing to its long‐term durability under real‐world environmental conditions. This durability was further confirmed through soiling tests that simulated the impact of airborne particulate matter on Coolcrete's albedo. Specifically, we assessed the effects of Saharan dust and carbon soot to replicate atmospheric conditions typical of sandstorms and urban pollution from heavy traffic. The results demonstrated that dust deposition led to less than a 1% reduction in solar reflectance, while soot caused a more noticeable decrease of ≈10% (see Figure S2E,F, Supporting Information). Importantly, in both cases, the original solar reflectance (≈0.95) was fully restored following a simple cleaning procedure using either forced air or gentle polishing (see Experimental Section). Similarly, the impact of both the soling test and the posterior cleaning process does not affect the coolcrete's emissivity in the AW (see Figure S2G,H, Supporting Information). Additional results confirming the durability of coolcrete (long‐term strength data, soiling cycle tests, and UV‐aging) are presented in Figure S3 (Supporting Information).

Furthermore, we would like to draw the attention to a key point of extreme importance for cement community; the embodied carbon of its fabrication. It is at this point that we note that the production of coolcrete has an extremely low upfront CO_2_ footprint. Figure 2E displays a benchmarking study that compares the global warming potential (kg CO_2_ eq. per dm^2^) of our coolcrete samples with other radiative cooling materials. The comparison is made for a tile fabricated with our coolcrete and regular OPC concrete tiles with several state‐of‐the‐art radiative cooling coatings. The methodology is detailed in N. Adams et al.,^[^ ^36^ ^]^ though its fundamentals are briefly explained in the Methods. As can be seen, coolcrete's family CO_2_ footprint (0.14–0.20 Kg CO_2_ eq.dm^2^) excels in the benchmarking analysis, comparing well with the values of a regular concrete tile (0.11 Kg CO_2_ eq.dm^2^) and being essentially equal to the tile with the hierarchically porous polymeric coatings (0.16–0.19 Kg CO_2_ eq.dm^2^) proposed by Mandal et al.^[^ ^12^ ^]^ A complete benchmarking analysis covering other environmental aspects is disclosed in Table S1 and Figure S4 (Supporting Information). Finally, price and cost are crucial factors for real‐world implementation in the construction market. A basic estimation, based on typical prices of key raw materials (zeolites at ≈250 € ton^−1^ and CaO at ≈90 € ton^−1^), indicates low production costs of less than 0.25 € m^−^^2^ for a 1 mm concrete layer. This cost remains highly economical and competitive—slightly below the estimated values for roll‐to‐roll radiative polymeric films ($0.35–‐$0.50 m^−^^2^)^[^ ^37^ ^,^ ^38^ ^]^ and cementitious coatings ($0.43 m^−^^2^)^[^ ^26^ ^]^—especially when considering the annual energy savings and CO_2_ reduction benefits it offers (see the Building and CO_2_ Saving section).

### 2.3 Outdoor Experiments

**Figure 3:**
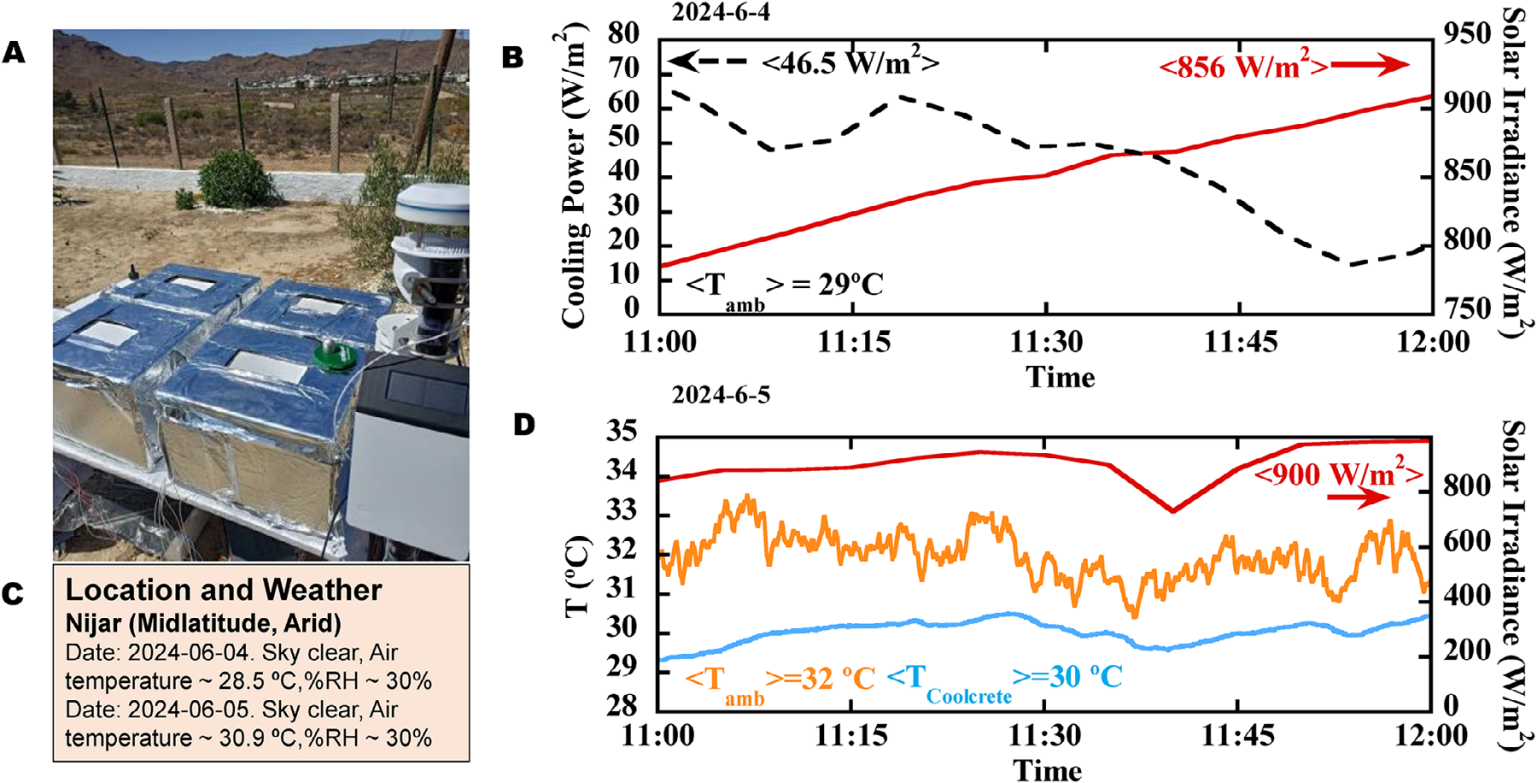
Set‐up and outdoor temperature measurements. A) Set‐up used for the measurements. B) Meteorological information of the days of measurement (data from 08:00 to 19:00). C) Cooling power (black dashed line) measured from 11:00 to 12:00 on the 2024‐06‐04. The solar irradiance is indicated by a solid red line. D) Temperature tracking from 11:00 to 12:00 on the 2024‐06‐05. The ambient and coolcrete's temperatures are shown by orange and blue lines, respectively. The measured solar irradiance is displayed by a red line.

Apart from lab‐scale measurements, we proved the radiative cooling performance of our coolcrete in a two‐day experimental campaign in Níjar, Spain (36.9667° N latitude and 2.2167° W longitude). Níjar represents a desertic climate with low humidity and high temperatures and sun irradiances (see **Figure** **3**A,B).

On the first day of measurements (2024/06/04) the cooling power of coolcrete was tracked with the help of an automatized feedback‐controlled heating system (see Experimental Section; Figure S5, Supporting Information). Figure 3C displays the data from 11:00 to 12:00 (local time), where our sample got positive radiative cooling power of ≈45 W m^−2^ under solar irradiances of ≈850 W m^−2^. It is worth noting that these values largely exceed the values of conventional concretes (typically between −300 and −600 W m^−2^).^[^ ^21^, ^22^ ^]^ The whole measurement window (08:00–19:00) (Figure S6A–C, Supporting Information) also revealed average promising average cooling powers above 30 W m^−2^ under average sun irradiances of 744 W m^−2^.

On the second day (2024‐06‐05), we followed the protocol employed by Mandal et al.,^[^ ^12^ ^]^ Two types of measurements were taken consecutively. One devoted to monitor the subambient temperatures (from 08:00 to 12:00) and a second one which measured the cooling power (from 12:00 to 19:00). In terms of the subambient temperatures, we found that the coolcrete sample remained clearly below the ambient air temperature during the whole morning (Figure S6B, Supporting Information), averaging a temperature drop of 4.1 °C under average solar irradiances of ≈625 W m^−2^. During the last hour of temperature measurement (Figure 3D), coolcrete reached temperatures ≈2°C lower than ambient under solar irradiances averaging 900 W m^−2^ and peaking values as high as 985 W m^−2^ (see Figure 3D). We emphasize that this subambient performance was achieved under very high ambient temperature (32°C on average) and without any convection shield. Afternoon, the experimental setup was changed to record the cooling powers, finding values in quite agreement with the ones measured on the preceding day. The whole measurement window is displayed in Figure S6D–F (Supporting Information).

To evaluate the radiative cooling performance of coolcrete in a climate completely different from that of Níjar, a 12‐day experimental campaign was conducted in San Sebastián from 2025‐07‐24 to 2025‐08‐04. Unlike Níjar, San Sebastián has a highly humid climate, with fluctuating temperatures and typically cloudy, windy, and rainy conditions. The results (see Figure S7, Supporting Information) again confirmed coolcrete's ability to maintain sub‐ambient temperatures during the central hours of the day, at times reaching up to ≈7 °C below the ambient air temperature. As expected, the experiment also showed that this sub‐ambient cooling capacity decreases under high humidity (above 80 %) and low ambient temperature conditions.

### 2.4 Geoclimatic Study

**Figure 4:**
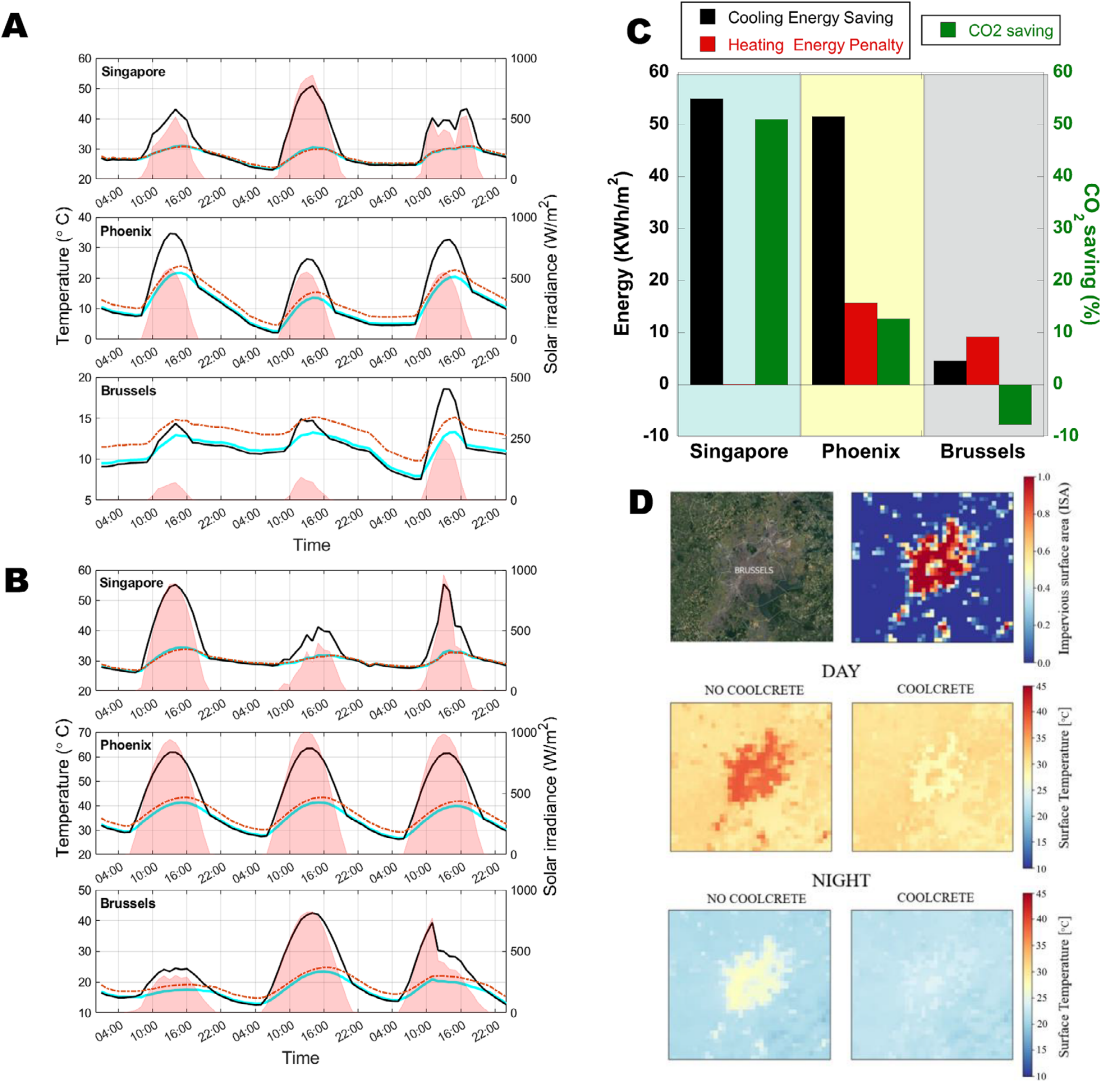
Modeling temperature reduction, cooling power in different climatic areas, and the impact on Urban Heat Island. A) Simulated steady‐state temperature around winter and B) summer solstices in Singapore, Phoenix, and Brussels. C) Building cooling energy saving, heating energy penalty, and CO_2_ saving for a mid‐rise building in Singapore, Phoenix, and Brussels. D) Combating the Urban Heat Island Effect. Urban temperature reduction in Brussels during the hot wave period of August 2019.

Based on the experimental values obtained in Nijar, a theoretical energy balance was conducted (see Experimental Section), showing that the measured cooling powers were consistent with a relatively high non‐radiative heat transfer coefficient (h_c_ ≈20 W m^−^^2^ K; see Experimental Section; Figure S8A–D, Supporting Information), which was expected due to the absence of a cover shield. Later, the study was extrapolated to different climatic scenarios by computational means following the methodology addressed in^[^ ^35^ ^]^ and briefly explained in the Methods. In particular, we investigated three cities, covering representative cases like Singapore for a humid climate, Phoenix for hot steppe, and Brussels for a continental climate with mild summers and cool winters. To provide a clearer understanding of the results and explore the relationship between net cooling power and meteorological variables, we focused on two 72 h periods centered around the winter and summer solstices (Figure 4A,B, respectively). For comparison purposes, the performance of a conventional OPC cement paste is also analyzed.

As observed, coolcrete remains consistently cooler than the ambient air across the three climates, regardless of the season. However, the temperature difference between coolcrete and both the ambient air and OPC sample varies depending on the specific case. In Singapore, the temperature difference between coolcrete and the ambient air is minimal. This is primarily due to the high relative humidity, which makes the atmosphere more opaque and reduces the effectiveness of passive radiative cooling. Despite this, given Singapore's overall mild temperatures, coolcrete still proves advantageous compared to OPC cement paste, which can reach temperatures exceeding 20 °C above the ambient air. In desert climates, such as Phoenix, coolcrete also stays below ambient temperatures during both winter and summer. In contrast, OPC cement paste can reach temperatures up to 10°C above ambient during solar irradiance peaks in winter and as much as 20 °C above ambient in summer. Such dry climate conditions, similar to those in Nijar, Spain, are particularly well‐suited for leveraging the cooling potential of coolcrete. In Brussels, the meteorological contrast between the cold winters and mild summers is more pronounced. During winter, our coolcrete steadily remains 2–3 °C below the per se cold ambient temperature (below 10 °C). The OPC sample is more sensitive to the sun irradiance, and its temperature is capable of approaching the ambient temperature during the solar irradiance peaks. Under these cold conditions, priority should be paid to heating rather than cooling, and the passive radiative cooling capacity of our coolcrete is surely undesirable. On the other hand, during summer, the temperature of coolcrete is just slightly lower (≈2.3 °C) than the temperate ambient temperatures, in clear contrast with OPCs, which can exceed 40°C during the central hours of the day.

### 2.5 Building Energy and CO_2_ Saving

On the basis of the abovementioned climatic scenarios, dynamic building simulations were conducted with EnergyPlus^[^ ^39^ ^]^ to estimate the annual net energy demands for heating and cooling of a mid‐rise building (Figures 4C; S9, Supporting Information). For all three cities, the application of coolcrete leads to a significant saving in cooling demand, with decreases of 4.56 kWh m^−^^2^ in Brussels, 51.59 kWh m^−^^2^ in Phoenix, and 55.01 kWh m^−^^2^ in Singapore. These cooling energy savings directly translate into electricity cost reductions. Assuming a conservative residential electricity rate of 0.15 € kWh^−1^, a building in Brussels could save 0.6 € m^−^^2^ annually, while buildings in Phoenix and Singapore could achieve savings of 7.73 € m^−^^2^ and 8.25 € m^−^^2^, respectively.

The reduction in cooling demand, however, is also accompanied by an increase in net heating demand (the so‐called winter penalty^[^ ^40^ ^]^) for Brussels and Phoenix, as these cities require heating during winter months. The net heating demand increases by 14.13 kWh m^−^^2^ in Brussels and by 15.72 kWh m^−^^2^ in Phoenix. Singapore, by contrast, experiences no heating demand year‐round, resulting in no associated heating penalty (Table S2, Supporting Information). This net annual energy demand analysis can be converted into equivalent CO_2_ emissions (kg CO_2_ eq. m^−2^), just assuming that the heating system is based on a condensing gas boiler and the cooling system in air conditioning (see Experimental Section; Table 4). This simple analysis shows how the winter penalty in Brussels outweighs the potential CO_2_ eq. Savings offered by coolcrete in summer, leading to an annual increase of ≈7% in the associated gross emissions. This is primarily due to Brussels’ climatic profile, where heating is more dominant than cooling. The relatively low cooling demand means that the potential for summer savings is limited, while the increased heating demand in winter, combined with the higher CO_2_ emission factor for gas heating (0.27 kg CO_2_ eq. per kWh) compared to electric cooling (0.13 kg CO_2_ eq. per kWh), results in a greater net environmental impact. However, noticeable CO_2_ eq. Savings of ≈12% and 51% are expected in Phoenix and Singapore, respectively (Table S2, Supporting Information).

### 2.6 Urban Heat Island effect

Due to the dominant role of cement‐based materials in cities, our developed radiative cooling concrete could be unique for fighting against the UHI effect. We explored the potential impact of our engineered coolcrete on the UHI effect using the COSMO‐CLM mesoscale regional climate model^[^ ^41^, ^42^ ^]^ over the urban area surrounding Brussels (see Figure 4D and Experimental Section for further information). The simulation domain encompassed an area of 331 by 151 grid cells across Brussels, with a horizontal grid spacing of 1 km. The assignment of albedo and emissivity values to specific pixels was determined by the Impervious Surface Area (ISA) ratio. This ratio indicates the proportion of urban surface within a given grid cell, where an ISA value of 1 means that the pixel is entirely urban (see the top panel in Figure 4D). The study spanned from April 2019 to September 2019, though the case study centered on the pernicious heatwave that occurred from August 23 to August 27, 2019. During this extreme heat wave, the average surface temperature in Brussels downtown exceeded 40°C during central hours of the day (6:00–18:00), ≈8.5 °C higher than the rural surrounding (middle panel in Figure 4D). At night (18:00–6:00), urban areas maintained a surface temperature ≈25°C, ≈4 degrees higher than rural areas (bottom panel in Figure 4D). According to our simulations, a noticeable temperature reduction would have occurred in the center of Brussels by applying coolcrete to all roofs of the city, creating an urban cool island effect. The surface temperature in the downtown would have shown a reduction close to 10 °C during the day and ≈5 °C at night, and the temperature gradient between the urban and rural areas would have disappeared. Similar encouraging results can be found when analysing the temperature difference at a height of two meters above ground level, where people experience heat (see Figure S10, Supporting Information). Simulations with varying levels of PMC roof coverage in Brussels show that temperature reductions diminish sharply as coverage decreases (Table S3, Supporting Information). Notably, halving the coverage results in significantly less than half the cooling, especially for 2‐meter air temperature, indicating a non‐linear relationship. This suggests that partial implementation yields limited impact, and high coverage is essential for meaningful UHI mitigation. When full coverage is not feasible, additional cooling strategies should be considered.

## 3 Conclusion

In summary, upgrading buildings, civil infrastructure, and urban areas to combat global warming is a worldwide challenge. Inspired by Roman mortars, we fabricated a scalable PDRC concrete (coolcrete) compatible with regular concrete technology that can revolutionize the very conception of concrete and how modern cities can be designed and planned. In this regard, our engineered coolcrete integrates a modern, sophisticated technology (radiative cooling technology) into an ancient and simple material like a Roman mortar. Durability is a standout feature of Roman mortars, and the same holds true for coolcrete. Its hydrophobic surface resists waterborne dirt, ensuring sustained performance and reliability under real‐world environmental conditions, outperforming alternative solutions in the literature. Besides, coolcrete's environmental impact is remarkably low. Benchmarking studies reveal a significantly reduced CO_2_ footprint compared to other radiative cooling materials, establishing it as a sustainable and eco‐friendly option. In terms of its photonic properties, coolcrete delivers exceptional performance, combining high solar reflectance (≈0.95) and long‐wave infrared (LWIR) emittance (≈0.91). Lab‐scale measurements and real‐time outdoor experiments validated its capability to achieve cooling powers of ≈45 W m^−^^2^ and sub‐ambient cooling of up to ≈2°C under average solar intensities of ≈850 and ≈900 W m^−2^, respectively. Computational models further predict significant energy and CO_2_ emission savings (≈51% per year in hot climates) and effective urban heat island (UHI) mitigation, with potential surface temperature reductions of ≈10°C during heatwaves. Given the overwhelming global reliance on concrete and cement‐based materials, coolcrete represents a transformative advance, offering scalable solutions for improving building energy efficiency and combating the increasingly recurrent Urban Heat Island effect. In the words of J. N. Munday,^[^ ^37^ ^]^ “Mitigating climate change is a tall task, and we are reaching a point where all the options should be on the table.” In this context, PDRC concretes like those presented here can play a significant role in addressing this monumental challenge.

## 4 Experimental Section

### Materials and Sample Processing

The materials utilized in this study included zeolite (Zeolite 4A) sourced from the IQE Group (Zaragoza, Spain), C_3_S obtained from Bonding Chemical (https://www.bondingchemical.com/), and white cement (BL II/A‐LL 52,5 R) from CIMSA (https://cimsacementos.es/). The activation was performed using calcium oxide (CaO), which was procured from Sigma–Aldrich (Germany).

The chemical composition of the zeolite was analyzed using X‐ray fluorescence spectroscopy (XRF), indicating that it primarily consists of SiO_2_, Al_2_O_3_, and Na_2_O (16%) (see **Table** **1**).

**Table 1:** Chemical composition of the zeolite.

	SiO_2_	Al_2_O_3_	Fe_2_O_3_	MgO	CaO	Na_2_O	K_2_O	TiO_2_	P_2_O_5_	SO_3_	LOI
**zeolite**	30.57	27.14	0.01	0.17	0.05	16.33	0.03	0.02	LD	LD	22.45

The preparation of samples using the hot mixing method involved combining the powdered starting materials, including zeolite, C_3_S, or white cement (WC), according to the mixture presented in **Table** **2**. Calcium oxide (CaO) was used as the activator and added to the powder mixture at a 2.5:1 ratio. Water was incorporated at a liquid‐to‐solid (L/S) ratio of 0.5. The mixtures were initially mixed for 2 min at 200 rpm, followed by 1 min at 950 rpm to ensure uniform hydration and optimal workability.

**Table 2:** Coolcrete family. Composition of the synthesized samples.

Sample ID	Aluminosilicate Source			CaO	Z/Ca	Water	L/S
	Zeolite	C3S	WC				
Coolcrete	100			40	2.5	70	0.5
Coolcrete/C3S	75	25		40	2.5	70	0.5
Colcrete/WC I	75		25	40	2.5	70	0.5
Coolcrete/WC II	50		50	40	2.5	70	0.5

The resulting mixtures were poured into molds and covered with plastic bags. After 24 h, the samples were demolded and cured in water for 28 days to complete the setting and curing process.

### XRD Analysis

Mineralogical analysis was performed using X‐ray diffraction (XRD) on a PANalytical Xpert diffractometer. This system was equipped with a copper tube (lCuKamedia = 1.5418 Å, lCuKa1 = 1.54060 Å, and lCuKa2 = 1.54439 Å), a vertical goniometer with Bragg‐Brentano geometry, a programmable divergence slit, an automatic sample exchanger, a secondary monochromator, and a PixCel detector. These features were chosen to enhance the precision and accuracy of the diffraction measurements. The measurement conditions were set at 40 kV and 40 mA, covering a scan range from 5 to 80° 2ϴ. This range was selected to capture comprehensive data on the mineral phases present within the sample, facilitating a thorough analysis. For processing the diffractogram and identifying the various phases within the sample, the PANalytical X'pert HighScore software was employed. This software was used in conjunction with the PDF2 database from the International Centre for Diffraction Data (ICDD), enabling precise phase identification. The quantification of the X‐ray diffraction data was carried out using the TOPAS software.

### FTIR Experiments

Fourier Transform Infrared (FTIR) experiments were carried out by means of a Jasco 6300 spectrometer in the mid‐infrared range. Measurements were conducted at ambient temperature (assumed to be 25 °C, unless otherwise stated). In order to minimize errors due to background and lamp intensity variations, background runs were recorded just before every sample measurement. The vibrational absorption spectra of the samples were recorded in an Attenuated Total Reflectance (ATR) configuration by means of a single reflection diamond ATR from Specac, equipped with a N_2_ purge. Samples were ground to obtain fine powders with a mesh size smaller than 45 µm and directly placed on top of the diamond prism. Pressure was then applied by screwing the anvil until the absorption peak intensity reached a maximum value. The reflectivity of the samples was determined by means of a gold‐coated 12° integrating sphere in downward configuration equipped with an MCT detector from PIKE. For these measurements, samples consisted of either solid pieces or fine‐grained powders, which were inserted into the sphere according to manufacturer specifications.

To accurately calculate the average reflectance over the Atmospheric Window (RAW), the following equation was employed:

(2) $$R_{AW}=\frac{\int_{8}^{13}R\left(\lambda \right)I_{BB}\left(\lambda \right)d\lambda }{\int_{8}^{13}I_{BB}\left(\lambda \right)d\lambda }$$

where the integration limits are fixed at 8 and 13 µm, which correspond to the boundaries of the AW, R(λ) is the measured reflectance at wavelength λ, and, *I_bb_* is the spectral blackbody irradiance.

(3) $$I_{bb}\left(T,\lambda \right)=\frac{2hc^{2}}{\lambda^{5}}\frac{1}{e^{\frac{hc}{\lambda k_{B}T}}-1}$$

With k_B_ the Boltzmann constant, h Planck's constant, c the speed of light, T the temperature, and λ the wavelength.

As customary in the state of the art, the emissivity in the AW (e_AW_) will be calculated as 1‐R_AW_ assuming T = 25°C.

### Solar Spectral Reflectance Measurements

The sun reflectance of the samples was characterized using a JASCO V770 UV–vis–NIR spectrometer equipped with a JASCO ILN‐925 UV–Vis/NIR Integrating Sphere. Measurements were conducted over a spectral range of 190–2700 nm, with a data interval of 1 nm, providing high‐resolution spectral information.

To accurately calculate the average reflectance over the solar spectrum (R_sun_), the following equation was employed:

(4) $$R_{\mathrm{sun}}=\frac{\int_{0.25}^{2.5}R\left(\lambda \right)I_{\mathrm{sun}}\left(\lambda \right)d\lambda }{\int_{0.25}^{2.5}I_{\mathrm{sun}}\left(\lambda \right)d\lambda }$$

where R(λ) is the measured reflectance at wavelength λ, and I_sun_(λ) is the solar irradiance. The solar irradiation data was obtained from the ASTM G173 Global Solar spectrum, ensuring standardized and reliable results.

### Porosity Measurements

Mercury intrusion‐extrusion porosimetry was conducted on the family of coolcrete samples using the AutoPore V porosimeter from Micromeritics to determine pore size distribution and volume. This analysis was performed at a maximum pressure of 33,000 psi to ensure comprehensive intrusion across a wide range of pore sizes. Prior to analysis, the sample underwent a controlled drying process at 110°C under vacuum conditions for 4 h. This step ensured the removal of any residual moisture and volatile substances that might affect the porosity assessment. The parameters configured for mercury during the analysis included a surface tension of 480 erg cm^−^^2^ and a contact angle of 130°, critical for accurate pore size determination using Washburn's equation.

### Mechanical Properties

The mechanical properties were assessed by compressive and flexural strength measurements, at 28 and 90 days, and they were carried out under a Tester Ibertest Press. All the strength data reported correspond to the average of six replicate specimens cast from the same batch.

### Confocal Raman Imaging

Raman spectroscopy measurements were conducted using a Renishaw inVia confocal Raman microscope. The system was equipped with a 633 nm laser and configured with an 1800 l mm^−1^ grating, providing high spectral resolution. Calibration of the spectrometer was performed using the 520 cm^−1^ peak of a silicon wafer standard. A Leica 100x microscope objective was employed to focus the laser beam onto the sample surface. The laser power was set to 100% to maximize signal intensity. For data acquisition, a scan area of 40 × 30 µm was selected, with a lateral resolution of 0.25 µm. A total of 16,000 individual spectra were collected, with an exposure time of 3 s per point. Data acquisition and processing were controlled using the Renishaw WiRE software package.

### Hydrophobicity

The water contact angle (WCA) of distilled water droplets on the surfaces of the sample was measured using an optical tensiometer equipped with a UI‐122XLE‐M camera from IDS. This analysis aimed to evaluate the hydrophilic or hydrophobic characteristics of the sample surface, which were critical for understanding and predicting its interaction with moisture in various applications. A droplet of 2 µL distilled water was accurately deposited onto the surface of each specimen. The droplet was allowed to achieve equilibrium, thus ensuring stable measurement conditions. Upon reaching equilibrium, an image of the water droplet was captured using the UI‐122XLE‐M camera integrated within the optical tensiometer system. The WCA was calculated utilizing the ellipse fitting method in the SCA20 measurement software, which fits an ellipse to the contour of the droplet to determine contact angles accurately. For each specimen, measurements were conducted with three droplets placed at three distinct points on the surface to ensure accuracy and account for any surface heterogeneity. The average WCA values were then calculated and reported, providing a reliable assessment of the surface's wetting properties.

### Soiling Resistance Tests

The soiling resistance evaluation followed a protocol inspired by ASTM D7897‐18, in which soiling agents were dispersed in distilled water, applied to the test surface, dried, and then characterized. To replicate the typical composition found in Saharan sand, 2.3 g of a mixture containing clays (50%), quartz (30%), calcite (10%), Fe_2_O_3_ (5%), and calcium sulfate (5%) was dispersed into 1.0 L of distilled water and mixed with a vortex mixer at 2000 rpm for 2 min until obtaining a stable mineral dust suspension. Likewise, carbon black (1.37 g) was dispersed into 1.0 L of distilled water and shaken until forming a stable soot suspension. The suspensions were sprayed uniformly onto coolcrete surfaces to reach a total dry concentration of 300 and 0.125 µg cm^−2^ for Saharan dust and soot, respectively. The soiled samples were dried under a 500 W halogen lamp for 15 min prior to measurements. Two cleaning procedures were carried out: soft cleaning using an air gun and hard cleaning by polishing the surface with a 400‐grit diamond pad.

### UV Aging Tests

Samples were placed in a Photoreactor m2 (PennPhD) equipped with a LED module emitting at 365 nm, consisting of four LEDs each with a power of 1.1 W (total power of 4.4 W) and releasing 17.5 W m^−2^ over the sample. The reactor was operated at a controlled temperature of 30 °C throughout the experiments. Samples were exposed to accelerated UV irradiance for 24 h. The irradiance level and exposure time were selected to achieve a total UV dosage equivalent to approximately half a year of Florida sunshine exposure, corresponding to an annual UV dose of ≈275 MJ m^−1^.

### Cradle‐to‐Gate Life Cycle Assessment—Goal and Scope Definition

The cradle‐to‐gate environmental impact of the coolcrete mixtures was assessed according to the A1–A3 modules specified in the EN15804+A2 standard.^[^ ^43^ ^]^ The impact assessment encompasses the system boundaries from raw material extraction (A1) through to production processes (A3). Inputs include the required energy and auxiliary materials for manufacturing, as well as known direct emissions. Cleaning, maintenance, labor, and transport impacts were excluded. The environmental impact was evaluated per declared unit of 1 dm^2^, allowing for a direct comparison with the environmental impact of ten established radiative cooling materials, evaluated using identical system boundaries.^[^ ^36^ ^]^ A comparative analysis of the environmental impact was made of the new material with current state‐of‐the‐art radiative cooling materials and a standard concrete roof tile.

### Life Cycle Inventory

The thickness of coolcrete was set at 0.5 cm. The quantities of the various components in the coolcrete compositions are listed in **Table** **3**. A lab mixer was assumed for the production process, with an energy consumption of 0.00059 kWh as explained in.^[^ ^36^ ^]^ The Ecoinvent v3.6 generic database was used for the inventory (Table 3) of the material and process records, adhering to allocation rules in the cut‐off system model.^[^ ^43^ ^]^ European data records were used where available, with global records as alternatives when necessary.

**Table 3:** Life cycle inventory. Life cycle inventory of coolcrete mixtures using the generic Ecoinvent v3.6 database.

	Component	Amount (mass percentage)	Ecoinvent v3.6 Record
COOLCRETE	Zeolite	37.521 g (48%)	Zeolite, powder {RER}| zeolite production, powder | Cut‐off, U
	CaO	15.008 g (19%)	Quicklime, milled, packed {RoW}| market for quicklime, milled, packed | Cut‐off, U
	Water	26.265 g (33%)	Tap water {RER}| market group for tap water | Cut‐off, U
	Energy (mixer)	0.00059 kWh	Electricity, medium voltage {Europe without Switzerland}| market group for electricity, medium voltage | Cut‐off, U
COOLCRETE /C3S	Zeolite	29.206 g (36%)	Zeolite, powder {RER}| zeolite production, powder | Cut‐off, U
	CaO	15.576 g (19%)	Quicklime, milled, packed {RoW}| market for quicklime, milled, packed | Cut‐off, U
	Water	27.529 g (33%)	Tap water {RER}| market group for tap water | Cut‐off, U
	C3S	9.735 g (12%)	Cement, Portland {Europe without Switzerland}| cement production, Portland | Cut‐off, U
	Energy (mixer)	0.00059 kWh	Electricity, medium voltage {Europe without Switzerland}| market group for electricity, medium voltage | Cut‐off, U
COOLCRETE /WC I	Zeolite	29.206 g (36%)	Zeolite, powder {RER}| zeolite production, powder | Cut‐off, U
	CaO	15.576 g (19%)	Quicklime, milled, packed {RoW}| market for quicklime, milled, packed | Cut‐off, U
	Water	27.529 g (33%)	Tap water {RER}| market group for tap water | Cut‐off, U
	WC	9.735 g (12%)	Cement, Portland {Europe without Switzerland}| cement production, Portland | Cut‐off, U
	Energy (mixer)	0.00059 kWh	Electricity, medium voltage {Europe without Switzerland}| market group for electricity, medium voltage | Cut‐off, U
COOLCRETE / WC II	Zeolite	20.236 g (24%)	Zeolite, powder {RER}| zeolite production, powder | Cut‐off, U
	CaO	16.189 g (19%)	Quicklime, milled, packed {RoW}| market for quicklime, milled, packed | Cut‐off, U
	Water	28.331 g (33%)	Tap water {RER}| market group for tap water | Cut‐off, U
	WC	20.236 g (24%)	Cement, Portland {Europe without Switzerland}| cement production, Portland | Cut‐off, U
	Energy (mixer)	0.00059 kWh	Electricity, medium voltage {Europe without Switzerland}| market group for electricity, medium voltage | Cut‐off, U

Life cycle impact assessment: Both the climate change impact, measured in kg CO_2_ eq., and the single‐score environmental impact (SSEI), expressed in mPt, were analyzed to identify potential discrepancies between indicators. The single score was calculated by applying normalization and weighting factors from the European Commission's Product Environmental Footprint (PEF) methodology.^[^ ^44^ ^]^

### Outdoor Test

The experimental procedures were conducted in Níjar, Spain, located at 36.9667° N latitude and 2.2167° W longitude, at an altitude of ≈355 meters above sea level. Prior to field experiments, preliminary indoor tests were carried out on both samples and the measurement box in the laboratory using a 500W halogen lamp. These tests helped validate the experimental setup and provided initial performance data under controlled conditions (see Figure 1A). Figure S4 (Supporting Information) illustrates the setup used for measuring cooling power. A thermal box was constructed to house a sample measuring 150 × 80 × 5 mm. The box itself was constructed from Expanded Polystyrene (EPS) and measured 250 × 250 × 320 mm. It was internally filled with polyethylene foam to enhance insulation. The sample measurement slot was centrally located at the top of the box. A Kapton heater with a thickness of 0.2 mm and dimensions matching the sample size (15 × 8 cm) was utilized. This heater was sandwiched between a Polyisocyanurate board, which prevented heat dissipation, and a copper plate of identical size, which enhanced thermal contact and distributed heat effectively to the sample. The sample was positioned level with the box top, ensuring uniform exposure. The temperature monitoring system incorporated two PT100 sensors. One sensor was positioned between the copper plate and the sample to ensure accurate temperature readings of the sample, while the other sensor, measuring ambient temperature, was placed next to the measurement box at the same height as the sample and shielded from direct sunlight to obtain a reliable setpoint for the heater's operation. During the experiment, the Kapton heater was activated, and a feedback control program ensured that the sample temperature was maintained equal to the ambient temperature. To minimize conductive heat loss from the ground, the thermal box was elevated one meter above ground level on a supportive table. Environmental conditions were measured using specialized equipment positioned adjacent to the measurement boxes. Wind speed was recorded using an ATMOS22 sensor by METER, while relative humidity (RH) was measured using an ATMOS 14 sensor, also by METER. Solar power radiation was measured using a pyranometer (APOGEE SP510) placed on top of the measurement box, capable of measuring in the spectral range of 385–2105 nm.

### Geoclimatic Study

The theoretical energy balance for radiative cooling materials (at temperature *T_s_*) facing the sky and surrounded by ambient air at temperature *T_amb_* is given by Equation (5):

(5) $$\begin{array}{ccc} P_{cool}\left(T_{s}\right) & = & P_{rad}\left(T_{s}\right)-P_{atm}\left(T_{amb}\right)-P_{sun}\\ & & -P_{cond+conv}\left(T_{s},T_{amb}\right) \end{array}$$

where *P_cool_* is the net cooling power calculated by subtracting the absorbed solar irradiation power (*P_sun_*), the absorbed power from the surrounding atmosphere (*P_atm_*), and the device's heat convection and conduction environment (*P*_*cond* + *conv*_) from the thermal power radiated from the material surface (*P_rad_*). The calculation of these terms is shown in Equations ((6), (7), (8), (9)):

(6) $$P_{rad}\left(T_{s}\right)=2\pi \int_{0}^{\frac{\pi }{2}}cos\theta sin\theta d\theta \int_{\lambda_{1}}^{\lambda_{2}}I_{bb}\left(T_{s},\lambda \right)\varepsilon \left(\lambda ,\theta \right)d\lambda$$

(7) $$\begin{array}{ccc} P_{atm}\left(T_{amb}\right) & = & 2\pi \int_{0}^{\frac{\pi }{2}}cos\theta sin\theta d\theta \\ & & \times \int_{\lambda_{1}}^{\lambda_{2}}I_{bb}\left(T_{amb},\lambda \right)\varepsilon \left(\lambda ,\theta \right)\varepsilon_{atm}\left(\lambda ,\theta \right)d\lambda \end{array}$$

(8) $$P_{cond+conv}\left(T_{s},T_{amb}\right)=h_{c}\left(T_{amb}-T_{s}\right)$$

(9) $$P_{sun}=\int_{0.25\;\mu m}^{2.5\;\mu m}\varepsilon \left(\lambda \right)I_{sun}\left(\lambda \right)d\lambda$$

where, *I_bb_* is the spectral blackbody irradiance ($I_{bb}\left(T,\lambda \right)=\frac{2hc^{2}}{\lambda^{5}}\frac{1}{e^{\frac{hc}{\lambda k_{B}T}}-1}$), *h_c_* is a comprehensive non‐radiative heat transfer coefficient (conduction and external convection) and λ_1_ and λ_2_ are the lower and upper limits of the infrared region (2.5 and 16 µm in the present study). The angular dependence of the atmospheric emissivity was approximated as $\varepsilon_{atm}\left(\lambda ,\theta \right)=1-t{\left(\lambda ,0\right)}^{\frac{1}{cos\;\theta \;}}$, where *t*(λ, 0) is the atmospheric transmittance in the zenith direction. Lastly, *I_sun_* (λ) is the solar irradiation spectrum. To determine it for a given location, begin with measurements of total solar irradiance obtained from meteorological stations, which record the incident solar power over the full spectrum. Although these measurements provide a total irradiance value, they lack specific spectral information. To generate a detailed spectrum, the ASTM G‐173 reference spectrum was normalized and scale it to the measured total irradiance. This scaling redistributes the power across wavelengths, aligning with the spectral shape of the standard spectrum while reflecting the actual integrated solar power under the specific atmospheric and geographic conditions at the measurement site.

To evaluate and compare the potential of the examined cements for radiative cooling in various regions, three geographic locations were chosen: Singapore, Phoenix, and Brussels, exemplifying a tropical rainforest climate (Af), an arid desert (BWh), and a temperate climate zone (Cfb) following the Köppen‐Geiger climate classification.^[^ ^46^ ^]^

The climatological data (ambient temperature, relative humidity, precipitable water, solar irradiance, etc.) were obtained from the Meteonorm (v7.2) tool (Meteonorm, (n.d.). https://meteonorm.com/ (accessed November 12, 2024).) over the course of a year, with a 1 h step resolution, using an average of the weather data from the last 10 years at each site. These data were then used to calculate the normal atmospheric transmittance profiles of each geographic location using the software MODTRAN.^[^ ^47^ ^]^

To validate the climate modeling study, meteorological data collected during the experimental campaign were used to calculate the theoretical net cooling power. This calculation critically depends on the convection power generated by the temperature difference between the ambient and the device. Accurately modeling the external heat convection factor (which determines how effectively this temperature difference was converted into convection power) was challenging due to significant discrepancies among existing empirical models,^[^ ^8^ ^]^ which can substantially affect net cooling power calculations. This parameter was influenced by factors such as wind speed, surface inclination, temperature differences, and surrounding surface temperatures. For the study presented in Figure 4 of the main text, a fixed convection coefficient of 20 W m^−^^2^ K was adopted as a reasonable and sufficiently high value. Although the convection coefficient naturally varies with local climatic conditions, our observations in Níjar strongly support this choice.

In an extended analysis (Figure S6, Supporting Information), uncertainties were further examined by incorporating a temperature interval of ±0.5 °C to account for the heater's response delay. Under these conditions, two convection scenarios were considered, hc = 5 W m^−^^2^ K for a low convection case and hc = 20 W m^−^^2^ K for a higher convection case, providing a comprehensive evaluation of the model's sensitivity to convection variations. These results not only corroborate the validity of the main study's assumptions but also underscore the importance of accounting for such uncertainties in climate modeling applications.

### Energy Efficiency in Buildings

This study investigates the impact of coolcrete on the energy requirements for heating and cooling across various climates. The analysis involves comparing the net energy demands for heating and cooling of TECNALIA's KUBIK test building, representing a residential single‐house family, for Brussels, Phoenix, and Singapore. Dynamic building simulations were conducted using EnergyPlus software,^[^ ^39^ ^]^ version 24.2.0.

TECNALIA's KUBIK test building serves as th,e case study for this research. Typical weather data, from EnergyPlus, of Brussels, Phoenix, and Singapore, were utilized to assess coolcrete's performance under different climatic conditions and following the Köppen classification.^[^ ^46^ ^]^

The KUBIK building has a total usable floor area of 345 m^2^, spread across three levels (see Figure S7, Supporting Information). This preliminary analysis exclusively utilizes the building's geometry. A simplified one‐zone energy model was developed, incorporating the thermal mass of internal structural elements. In this investigation, internal heat gains from occupants, lighting, or equipment were disregarded. To isolate the impact of coolcrete, both the roof and walls were modeled as 20 cm‐thick concrete structures with an albedo of 0.35 and an emissivity of 0.9. Windows were assumed to be double‐glazed, with a thermal transmittance (U‐value) of 2.7 W m^−^^2^ K and a solar heat gain coefficient (g‐value) of 0.7. The geometry of the energy model is shown in Figure S7 (Supporting Information).

In the scenario with coolcrete, the traditional concrete roof was replaced with the newly developed material. Since coolcrete was derived from standard concrete, only key optical properties such as albedo and emissivity were adjusted. These values were updated to 0.95 and 0.91, respectively, reflecting the unique photonic characteristics of coolcrete.

To evaluate the building's heating and cooling energy demands, simulations were conducted using an ideal loads air system (with infinite capacity) to maintain the desired indoor temperatures specified for each city in **Table** **4**. Nighttime operation of heating and cooling systems was excluded. Hourly simulation data was utilized to analyse performance outcomes.

**Table 4:** Conditions for estimating the energy demands. Minimum and maximum indoor temperatures for the ideal heating and cooling system for Brussels (BRU), Phoenix (PHX), and Singapore (SG).

	Period	Time Interval	Min. and Max. Indoor Temperature
BRU & PHX	1^st^ of October – 31^st^ of March	07:00–23:00	Heating, T_min_ = 20°C
	1^st^ of April – 30^th^ of April	07:00–23:00	Heating, T_min_ = 20°C, Cooling, T_max_ = 25°C
	1^st^ of May – 30^th^ of September	07:00–23:00	Cooling, T_max_ = 25°C
SG	All year	07:00–23:00	Cooling, T_max_ = 25°C

The net energy demand was converted into equivalent CO_2_ emissions (kg CO_2_ eq.) using the Ecoinvent v3.6 database^[^ ^45^ ^]^ to estimate potential reductions in summer emissions and increases in winter emissions. The heating system was assumed to be a condensing gas boiler with emissions of 0.27 kg CO_2_ eq. per kWh of gas and an efficiency of 1.02. For cooling, a conventional air conditioning system with an efficiency of 3.01 was used, resulting in emissions of 0.13 kg CO_2_ eq. per kWh of electricity.

One important consideration was the spectral emissivity of photonic materials like coolcrete. These materials, including other reflective coatings, exhibit high emissivity in the atmospheric window. However, this spectral property cannot be directly modeled in EnergyPlus, as the software requires a single averaged emissivity value. A study by Yu and Chen^[^ ^49^ ^]^ examines the effect of using an averaged emissivity to simulate reflective coatings in EnergyPlus. Their research suggests that this simplification slightly overestimates cooling potential, which should be acknowledged when interpreting the findings of this study. All parameters and assumptions used in the building energy simulations are summarized in Table S4 (Supporting Information).

### Temperature Impact at the Level of Cities and Urban Heat Island Effect

The effect on urban temperatures by large‐scale application of coolcrete was assessed using regional climate modeling. The COSMO‐CLM mesoscale regional climate model,^[^ ^41^, ^42^ ^]^ which has been successfully applied at the km scale^[^ ^50^ ^]^ has been configured for a simulation focused on Brussels, Belgium. This model integration spans from April 2019 to September 2019, utilizing ERA5 data as the forcing at the lateral boundaries.^[^ ^51^ ^]^ The case study centers on a heatwave that occurred from August 23 to August 27, 2019, with the preceding months from April serving as the spin‐up period for the soil model TERRA‐ML. A bulk urban land surface scheme TERRA_URB^[^ ^52^, ^53^, ^54^ ^]^ was included in COSMO‐CLM to enhance the representation of urban physics.^[^ ^53^ ^]^ The simulation domain encompasses an area of 331 by 151 grid cells around Brussels, with a horizontal grid spacing of 1 km. Additionally, a sensitivity analysis was conducted to assess the impact of the domain size, confirming that the selected domain size was large enough to correctly simulate temperature.

The COSMO‐CLM simulations were conducted at a 1 km grid resolution, representing a practical trade‐off between spatial detail and computational feasibility. While this resolution does not resolve individual buildings or fine‐scale urban heterogeneity, it was suitable for capturing mesoscale urban heat island (UHI) patterns and city‐wide surface–atmosphere interactions, particularly when combined with urban canopy parameterizations like TERRA‐URB.^[^ ^41^ ^]^ Although finer‐scale processes such as shading and rooftop‐level mixing require high‐resolution microscale models,^[^ ^55^ ^]^ COSMO‐CLM remains a robust tool for assessing the regional UHI mitigation potential of coolcrete. A combined modeling approach, using regional models for large‐scale effects and microscale models like ENVI‐met or PALM for neighbourhood‐level detail, was therefore recommended.

To incorporate photonic properties into the COSMO‐CLM model using TERRA_URB, a single representative value was required. Consequently, the spectral albedo (α) and emissivity (ε) of coolcrete were averaged, with weights applied based on solar irradiance and atmospheric transmittance within the atmospheric window. This process yields a singular albedo and emissivity value that serves as input for the COSMO‐CLM model. For coolcrete, the average emissivity was 0.91, while the average albedo was 0.95.

The assignment of new albedo and emissivity values to specific pixels was determined by the Impervious Surface Area (ISA) ratio. This ratio indicates the proportion of urban surface within a given grid cell, where an ISA value of 1 signifies that the pixel was entirely urban. A map of the ISA value for the city of Brussels and the surrounding rural area is presented in Figure 4D. The albedo and emissivity for the urban fraction of each pixel were adjusted to reflect these new parameters. Pixels with an ISA greater than 0.5 were classified as dense urban (DU), whereas those with an ISA below 0.05 were categorized as rural.^[^ ^51^ ^]^ Based on this classification, a bounding box was established around the city, maintaining a DU to rural pixel ratio of 0.2. The urban heat island (UHI) effect was then assessed using this box, comparing the temperatures of DU areas to those of rural regions.

The Semi‐empirical Urban Canopy Parametrization (SURY) was utilized to incorporate photonic properties into the urban canopy.^[^ ^54^ ^]^ SURY translates 3D urban canopy parameters into bulk parameters. This approach enhances existing urban bulk land surface schemes by integrating canopy‐dependent urban physics, thereby accounting for the heterogeneity present in urban canopy parameters across the city.

The calculated bulk photonic properties for each simulation are summarized in **Table** **5**. The COSMO‐CLM model was executed twice. The first run serves as the reference (CTL), utilizing the bulk albedo and emissivity without the application of coolcrete in the city. In the second run (PAR), the coolcrete was implemented across all urban roofs in Brussels. Lastly, to assess the influence of partial roof coverage with PMC on urban temperature mitigation, additional simulations were conducted using varying coverage ratios: 5%, 10%, 20%, 50%, 75%, and 100%. These scenarios were used to evaluate how different levels of implementation affect the reduction of the urban heat island (UHI) effect.

**Table 5:** Inputs for the bulk photonic properties. The bulk photonic properties of the two runs carried out with the COSMO‐CLM regional climate model.

Run	Bulk Albedo	Bulk Emissivity	Scenario
CTL	0.082	0.888	Control run without coolcrete
PAR	0.646	0.928	Coolcrete on all urban roofs

## Conflict of Interest

The authors declare no conflict of interest.

## Supporting information



Supporting Information

## Data Availability

The data that support the findings of this study are available from the corresponding author upon reasonable request.
