# Supplementary material for: A Modern Roman‐Inspired Concrete with Daytime Radiative Cooling Capacity
Source: Adv Sci (Weinh). 2025 Sep 12;12(47):e11691. doi: 10.1002/advs.202511691 (PMC12713017; doi:10.1002/advs.202511691)
Supplement: Supplementary file 1 — Supporting Information [file ADVS-12-e11691-s001.docx]

Supporting Information

A modern Roman-inspired concrete with Daytime Radiative Cooling Capacity

Jorge S. Dolado ^1,2*,^ Guido Goracci^1^, Ghizlane Moutaoukil^1^, Ridwan O. Agbaoye^1^, Miguel Beruete^3,4^, Alicia E. Torres-García^3^, Laura Carlosena^5^, Achutha Prabhu^6^, Jose A. Ibáñez^6^, Nick Adams^7^, Nicole van Lipzig^8^, Karen Allacker^7^

^1^ Centro de Física de Materiales (CFM) CSIC-UPV/EHU); San Sebastián, 20018, Spain.

^2^ Donostia International Physics Center (DIPC); San Sebastián, 20018, Spain

^3^ Department of Electrical, Electronic and Communications Engineering, Public University of Navarre

(UPNA); Pamplona, 31006, Spain.

^4^ Institute of Smart Cities (ISC), Public University of Navarre (UPNA); Pamplona, 31006 Spain

^5^ Department of Engineering, Public University of Navarra (UPNA); Pamplona, 31006, Spain

^6^ TECNALIA, Basque Research and Technology Alliance (BRTA), Derio 48160, Spain

^7^ KU Leuven, Faculty of Engineering Science, Department of Architecture,

Kasteelpark Arenberg 1 – box 2431, Leuven, 3001, Belgium

^8^ KU Leuven, Department Earth and Environmental Sciences, Celestijnenlaan 200E, 3001 Leuven,

Belgium

*Correspondence: j.dolado@ehu.eus

**This PDF contains**:

Figures S1-S10

Tables S1-S4

**Figure S1. Microstructure of coolcrete**. (**A)** Confocal Raman microscopy. Blue areas can be identified as zeolites and the red ones areas with large content of tobermorite-like and C-(A)-S-H phases. (**B)** XRD patterns for coolcrete and coolcrete_C3S samples. Z stands for zeolite, T for tobermorite (including CSH and CASH structures), Cc calcium carbonite, C Calcium Oxide, and C3S alite. The crystalline phases of coolcrete were quantified by using the TOPAS software, finding Z (51.33 %), T (21.05%), Cc (11.65%) and C (17.97%) . (**C)** Mercury Intrusion Porosity (MIP) measurements over coolcrete’s family; Log Differential vs pore size

 **Figure S2. Properties of Cooolcrete**. (**A)** Reflectance of the main phases appearing in coolcrete’s microstructure (zeolites and tobermorite) in comparison to the coolcrete’s reflectance. (**B)** Reflectance of the coolcrete’s family. (**C)** Photogram showing a water droplet over a coolcrete sample with a contact angle of 140º. Below, the evolution of a dye, rolling out of the surface of the coolcrete surface. (**D)** Compressive strength of the coolcrete’s family. For comparison, the minimum strength values of structural concretes such as C20 and C30 are also shown. (**E)** Reflectance of coolcrete in comparison to the reflecetance after a soling test with mineral dust and a posterior cleaning process. (**F)** Reflectance of coolcrete in comparison to the reflecetance after a soling test with soot and a posterior cleaning process. (**G)** IR absorbance in the AW of coolcrete in comparison to the one after a soling test with dust and a posterior cleaning process. **(H)** IR absorbance in the AW of coolcrete in comparison to the one after a soling test with soot and a posterior cleaning process.

**Figure S3. Durability of Cooolcrete**. (**A)** Reflectance of coolcrete after soiling cycle tests with soot. The cycles did not significantly reduce the reflectance compared to the initial soiling test (~10 % reduction), and cleaning consistently restored the original values.
**(B)** Reflectance of coolcrete after soiling cycle tests with dust. Similarly, the cycles caused less than a 5 % reduction in reflectance, and cleaning fully restored the original values.
**(C)** A sample cleaned after five soiling cycles with dust was subjected to UV aging. The solar reflectance of the sample remained unchanged after UV exposure. After the UV treatment, the hydrophobic capacity of the same sample was measured. Although the value (120°) was slightly lower than the original (140°), the hydrophobic character was preserved.
**(D)** Flexural and compressive strength of coolcrete at 28 and 90 days. As expected, coolcrete showed good compressive strength stability, maintaining values above the standards for masonry mortar applications.


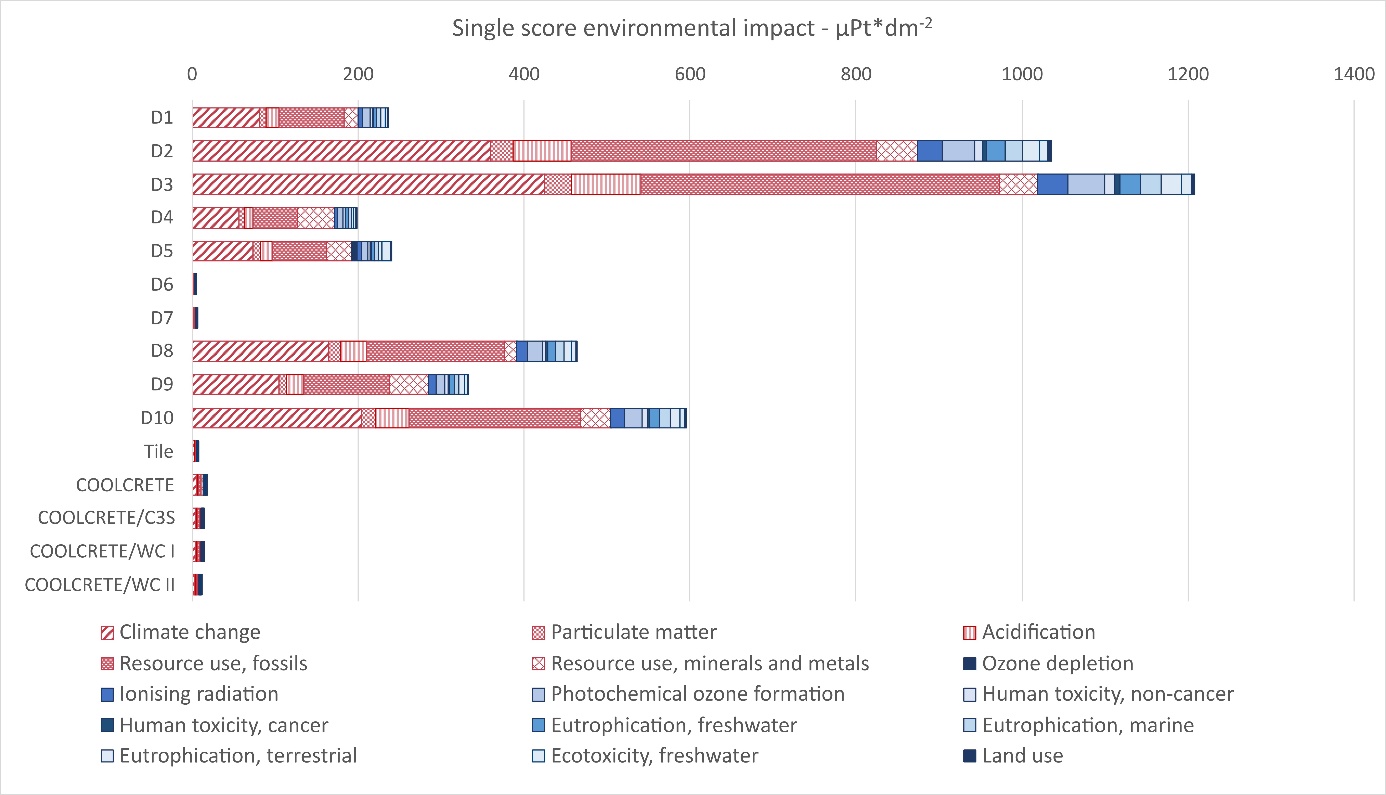


**Figure S4. Environmental benchmarking**. Single score environmental impact of coolcretre mixtures compared to the radiative cooling materials database from Ref (27) showing the absolute contribution of the various impact categories.

**
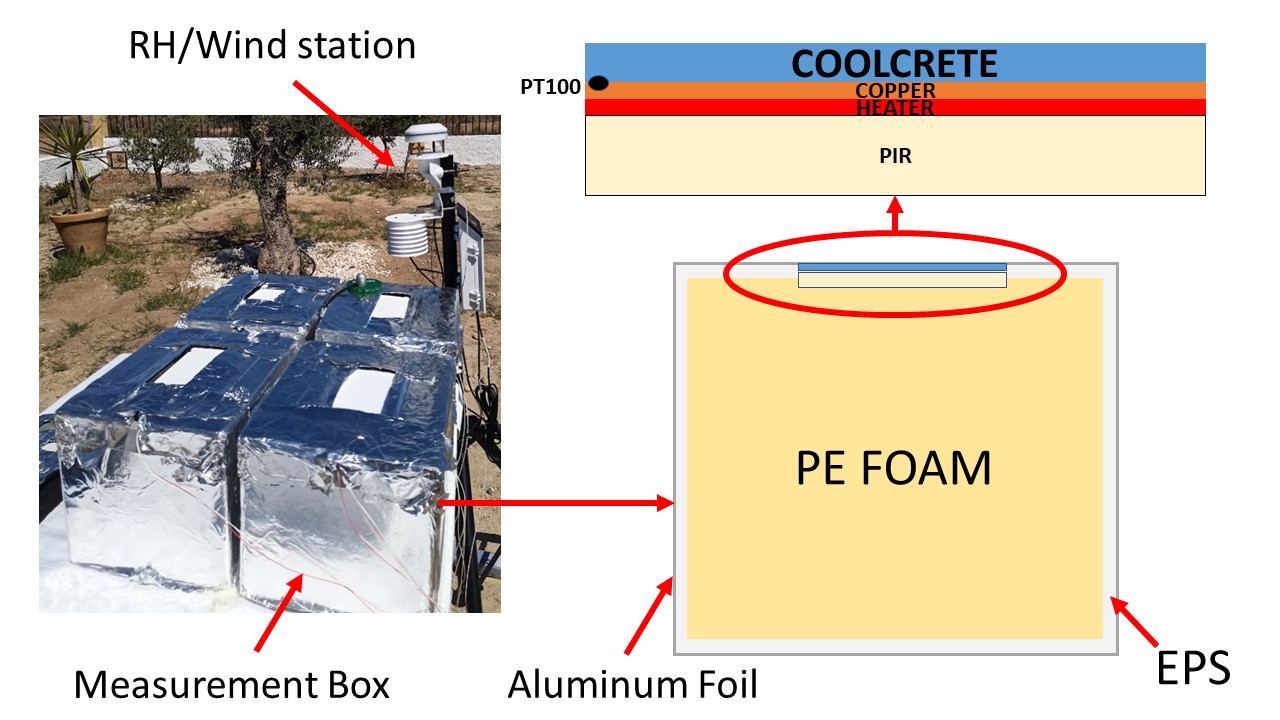
**

**Figure S5. Schematic illustration of the experimental set-up. Nijar:** A thermal box was constructed to house a sample measuring 150 × 80 × 5 mm. The box itself is constructed from Expanded Polystyrene (EPS) and measures 250 × 250 × 320 mm. It is internally filled with polyethylene foam to enhance insulation. The sample measurement slot is centrally located at the top of the box. A Kapton heater with a thickness of 0.2 mm and dimensions matching the sample size (150 × 80 mm) was utilized. This heater is sandwiched between a Polyisocyanurate board, which prevents heat dissipation, and a copper plate of identical size, which enhances thermal contact and distributes heat effectively to the sample. The sample is positioned level with the box top, ensuring uniform exposure. The temperature monitoring system incorporates two PT100 sensors. One sensor is positioned between the copper plate and the sample to ensure accurate temperature readings of the sample, while another sensor measures the ambient temperature, shielded from direct sunlight to obtain a reliable setpoint for the heater's operation. During the experiment, the Kapton heater was activated, and a feedback control program ensured that the sample temperature was maintained equal to the ambient temperature. To minimize conductive heat loss from the ground, the thermal box is elevated one meter above ground level on a supportive table. Environmental conditions, including wind speed and relative humidity (RH), are recorded using a mobile weather station. The experimental procedures were conducted in Níjar, Spain, located at 36.9667° N latitude and 2.2167° W longitude, at an altitude of approximately 355 meters above sea level. **San Sebastián**: Additional measurements were carried out on the rooftop of the Centro de Física de Materiales in San Sebastián-Donostia (43.3183° N, 1.9812° W) from July 24th to August 4th, 2025. For this experimental campaign, the setup was slightly modified to accommodate the new location and measurement requirements. The thermal box was reconfigured to allow measurement of a disc-shaped sample with a diameter of 40 mm and thickness of 5 mm. Since only temperature differences were monitored in this phase, the Kapton heater was removed from the setup, simplifying the configuration while maintaining the essential temperature sensing capabilities.

**Figure S6. Outdoor measurements in Nijar**. **A**, Continuous cooling power measurements (black line) on 2024-06-04 together with the solar irradiance (red line). **B**, Ambient temperature (orange) in comparison to the temperature of the sample (blue). As the heater was on, the sample’s temperature is forced to follow the ambient temperature. **C**, Relative humidity (blue line) and wind speed (green line) measured on 2024-06-04. **D**. Extended data of the measurements corresponding to the experiments on 2024-06-05. Initially (from 08:00 to 12:00) the heater was off, and the coolcrete’ sample temperature (blue line) was recorded in comparison to the ambient one (orange). After noon, the heater was on, and the cooling power (black line) was measured. The solar irradiance (red line) was continuously measured along the day. **E**, Temperature of coolcrete in comparison to the ambient temperature. From 12:00 onwards, both temperatures were forced to coincide. **F**, Relative humidity (blue line) and wind speed (green line) measured on 2024-06-05.

**Figure S7. Outdoor measurements in San Sebastián**. Continuous measurements from 2025-07-24 to 2025-08-04 of ambient air temperature and coolcrete surface temperature on the roof of the Centro de Física de Materiales (San Sebastián). Solar irradiance, humidity, and rainfall were also recorded. Symbols are included to illustrate the prevailing climatic conditions.

**Figure S8. Theoretical study**. Theoretical cooling power predictions compared with experimental measurements obtained in Nijar on two different dates. Panels **(A)** and **(B**) show data from 2024-06-04 and 2024-06-05, respectively, using an external convection coefficient of h_c_ = 5 Wm⁻²K⁻¹. Panels **(C)** and **(D)** present the corresponding results for the same dates assuming a higher convection coefficient of h_c_ = 20 Wm⁻²K⁻¹.

**Fig. S9. Geometry of the building.**  The case of study corresponds to Tecnalia’s KUBIK test building (<https://www.tecnalia.com/infraestructuras/edificio-experimental-kubik>). **(A)** South East facades. **(B)** North and West facades.

**Fig. S10. Two-meter temperature reduction analysis**. Daytime and nighttime average two-meter temperatures and two-meter temperature reduction for all the selected grid boxes in Brussels comparing simulations with and without coolcrete during the selected five-day heatwave.

**Table S1:** Comparison of the single score environmental impact and CO_2_ footprint of coolcrete mixtures to various radiative cooling materials from the database of Adams et al. (2024) (Ref. 27). The comparison includes standalone climate change impact (in kg CO_2_ eq/dm²) and the additional impact when incorporating a concrete roof tile as a supporting structure, where applicable.

| RC material | Single score environmental impact  [µPt/dm^2^] | Single score environmental impact with concrete roof tile  [µPt/dm^2^] | Climate change impact  [Kg CO_2_ eq/dm^2^] | Climate change impact  with concrete roof tile  [Kg CO_2_ eq/dm^2^] |
| --- | --- | --- | --- | --- |
| D1 | 234.30 | 240.57 | 3.12 | 3.23 |
| D2 | 1027.22 | 1033.49 | 13.84 | 13.95 |
| D3 | 1199.35 | 1205.62 | 16.29 | 16.40 |
| D4 | 196.79 | 203.06 | 2.19 | 2.30 |
| D5 | 239.11 | 245.38 | 2.84 | 2.95 |
| D6 | 3.77 | 10.04 | 0.05 | 0.16 |
| D7 | 6.03 | 12.30 | 0.08 | 0.19 |
| D8 | 460.71 | 466.98 | 6.36 | 6.47 |
| D9 | 330.84 | 337.11 | 4.03 | 4.14 |
| D10 | 591.38 | 497.65 | 7.87 | 7.98 |
| Tile | 6.27 | / | 0.11 | / |
| COOLCRETE | 16.94 | / | 0.20 | / |
| COOLCRETE  /C3S | 13.74 | / | 0.17 | / |
| COOLCRETE  /WC I | 13.74 | / | 0.17 | / |
| COOLCRETE  /WC II | 10.29 | / | 0.14 | / |

**Table S2.** **Building energy demand and CO_2_ footprint**. Difference in annual net energy demand [kWh/m^2^] for heating (gas) and cooling (electricity) with and without coolcrete in Brussels, Phoenix and Singapore, and associated gross emissions [kg CO_2_ eq. /m^2^] for heating and cooling with and without coolcrete in Brussels, Phoenix and Singapore (in brackets).

|  |  | **Energy use [kWh/m^2^] (Emissions [kg CO_2_ eq./m^2^])** | | **Delta energy use**  **(Delta emissions)** | | **Total delta emissions** | |
| --- | --- | --- | --- | --- | --- | --- | --- |
|  |  | no COOLCRETE | COOLCRETE | [kWh/m^2^]  ([kg CO_2_ eq./m^2^]) | [%] | [kg CO_2_ eq./m^2^] | [%] |
| BRU | heating | 153.51 (41.45) | 167.64 (45.26) | +14.13 (+3.82) | +9.21 | +3.22 | +7.63 |
|  | cooling | 5.84 (0.76) | 1.28 (0.17) | -4.56 (-0.60) | -78.06 |  |  |
| PHX | heating | 16.77 (4.53) | 32.49 (8.77) | +15.72 (+4.24) | +93.70 | -2.49 | -12.68 |
|  | cooling | 115.82 (15.12) | 64.23 (8.39) | -51.59 (-6.74) | -44.54 |  |  |
| SG | heating | 0.00 (0.00) | 0.00 (0.00) | 0.00 (0.00) | 0.00 | -7.18 | -51.01 |
|  | cooling | 107.85 (14.08) | 52.84 (6.90) | -55.01 (-7.18) | -51.01 |  |  |

**Table S3.** **Relationship between coolcrete** **coverage and temperature reduction** Average daytime (6 a.m. until 6 p.m.) and nighttime (6 p.m. until 6 a.m.) surface temperature (Ts) and two-meter temperature (T-2m) reduction in Brussels for different COOLCRETE covering fractions.

| **Coverage fraction** | **100%** | **75%** | **50%** | **20%** | **10%** | **5%** |
| --- | --- | --- | --- | --- | --- | --- |
| Nighttime Ts reduction | 3.35 | 2.28 | 1.39 | 0.51 | 0.23 | 0.11 |
| Daytime Ts reduction | 7.63 | 5.36 | 3.34 | 1.24 | 0.60 | 0.29 |
| Nighttime T-2m reduction | 2.24 | 1.48 | 0.85 | 0.28 | 0.12 | 0.05 |
| Daytime T-2m reduction | 1.36 | 0.80 | 0.46 | 0.16 | 0.07 | 0.03 |

**Table S4.** **Summary of building energy simulation parameters and assumptions used to evaluate COOLCRETE’s impact on heating and cooling energy demand.** The table outlines key input assumptions used in EnergyPlus simulations for a residential single-family case study (KUBIK test building) in Brussels, Phoenix, and Singapore. It includes building geometry, envelope properties, HVAC system characteristics, climate data, and operational settings. The parameters are chosen to isolate the influence of coolcrete optical properties on energy performance and CO₂ emissions.

| **Category** | **100%** |
| --- | --- |
| Building Characteristics | Type: Residential single-family house (KUBIK test building)  Floor area: 345 m²  Levels: 3  Energy model: Simplified one-zone model |
| Envelope | Roof & walls: 20 cm concrete  Regular concrete - albedo: 0.35, emissivity: 0.90  COOLCRETE - albedo: 0.95, emissivity: 0.91 |
| Windows | Double-glazed  U-value: 2.7 W/m²K  Solar Heat Gain Coefficient (g-value): 0.7 |
| HVAC systems | **Heating**: Condensing gas boiler  Efficiency: 1.02  Emissions: 0.27 kg CO₂/kWh  **Cooling**: Conventional air conditioning  COP: 3.01  Emissions: 0.13 kg CO₂/kWh |
| Climate Locations | Brussels, Phoenix, Singapore (typical year weather data from EnergyPlus) |
| Internal Gains | No internal heat gains from occupants, lighting, or equipment included |
| Additional Notes | Ideal loads air system (infinite capacity) used to isolate envelope effects |
